# Mining locus tags in PubMed Central to improve microbial gene annotation

**DOI:** 10.1186/1471-2105-15-43

**Published:** 2014-02-05

**Authors:** Chris J Stubben, Jean F Challacombe

**Affiliations:** 1 Bioscience Division, Los Alamos National Laboratory, Los Alamos, NM, USA

## Abstract

**Background:**

The scientific literature contains millions of microbial gene identifiers within the full text and tables, but these annotations rarely get incorporated into public sequence databases. We propose to utilize the Open Access (OA) subset of PubMed Central (PMC) as a gene annotation database and have developed an R package called pmcXML to automatically mine and extract locus tags from full text, tables and supplements.

**Results:**

We mined locus tags from 1835 OA publications in ten microbial genomes and extracted tags mentioned in 30,891 sentences in main text and 20,489 rows in tables. We identified locus tag pairs marking the start and end of a region such as an operon or genomic island and expanded these ranges to add another 13,043 tags. We also searched for locus tags in supplementary tables and publications outside the OA subset in *Burkholderia pseudomallei* K96243 for comparison. There were 168 publications containing 48,470 locus tags and 83% of mentions were from supplementary materials and 9% from publications outside the OA subset.

**Conclusions:**

*B. pseudomallei* locus tags within the full text and tables of OA publications represent only a small fraction of the total mentions in the literature. For microbial genomes with very few functionally characterized proteins, the locus tags mentioned in supplementary tables and within ranges like genomic islands contain the majority of locus tags. Significantly, the functions in the R package provide access to additional resources in the OA subset that are not currently indexed or returned by searching PMC.

## Background

The rapid growth of next generation sequencing and transcriptomic studies, particularly on the causative agents of infectious diseases, requires accurate genome annotations to confidently analyze the sequencing data and identify and compare functions, pathways and networks. There are many resources available for genome annotation and most rely on transferring annotations from model organism or protein family databases that vary greatly in content and quality [1]. For microbial genomes, there are very few model organism databases containing manual annotations based on experimental evidence in the current literature. Therefore, when microbial genomes are reannotated or new gene functions are identified by subsequent experiments, the new updates are rarely incorporated into public sequence databases.

Since the manual annotation of genomes using controlled vocabularies and evidence codes is a time-consuming task [2], text mining solutions that link evidence in the literature to annotations in genome databases are needed [3,4]. One recent example is text2genome, which extracts DNA sequences from PubMed Central (PMC) and maps them to model organism databases [5]. Significantly, this study was the first to mine text in supplementary files in the Open Access (OA) subset. The authors found DNA sequences in 20% of the OA articles and then requested permission to mine the full text from over 40 publisher websites (their progress and efforts over the last three years are documented on the UCSC Genocoding website at http://text.soe.ucsc.edu). A related project called pubmed2ensembl links millions of articles to thousands of genes from 50 eukaryotic species using six data sources containing gene to literature links [6].

Many other projects have shown that text mining improves the links between literature and biological databases such as the Protein Data Bank and Gene Expression Omnibus [7] or UniProt and the European Nucleotide Archive [8]. In this latter study, the authors noted the existence of accession number ranges but did not attempt to expand or quantify the regions. Many other tools have been developed to extract information from biological texts and are reviewed in [9-12]. Most of the text mining applications discussed in these reviews focus on innovative efforts to extract genes, functions and interactions from model eukaryotic organisms.

For microbial genomes with very few functionally characterized proteins, locus tags are often associated with structural and functional annotations in the literature. Structural annotations may include revised gene starts, novel genes or mobile regions based on either computational or experimental evidence. Functional annotations may include the assignment of new definitions, gene names and functions. Therefore, a typical role filled by model organism databases is to update annotations by linking genes to experimental evidence in the literature, and text mining tools are often used to assist in the process of manual curation [12]. Another option for curators is to use a full text database like PMC to search for articles citing a specific gene or locus tag in the full text. However, finding the locus tag within the article requires searching through the entire text and linked tables. To facilitate these types of automated searches, we developed an R package to mine locus tags from text, tables and supplements in the OA subset.

We demonstrate the capabilities of these tools by mining locus tags from ten microbial genomes. Our main objectives are to (1) improve access to structured data in order to extract locus tags from rows that are linked to column names, captions and subheadings, (2) identify locus tags pairs marking the start and end of a region and then list genes mentioned indirectly within the range, and (3) search for all locus tags in supplementary tables and publications outside the OA subset in *Burkholderia pseudomallei* K96243 for comparison. This comprehensive set of locus tags in *B. pseudomallei* is used to highlight deficiencies in current annotations and suggest future microbial gene mining efforts.

## Implementation

Searching for a single locus tag in PMC is straightforward, for example, enter “Rv3874” in the search box and this will return 135 articles (accessed Nov 5, 2013). These full text articles are part of two groups in PMC, an Open Access subset that are available for text mining and another set that are merely free to read. The OA subset are available as XML files that can be downloaded using automated queries to either the FTP site or Open Archives Initiative service and articles may be searched by adding the Open Access filter (Rv3874 AND open access[FILTER] returns 47 results).

**Figure 1:**
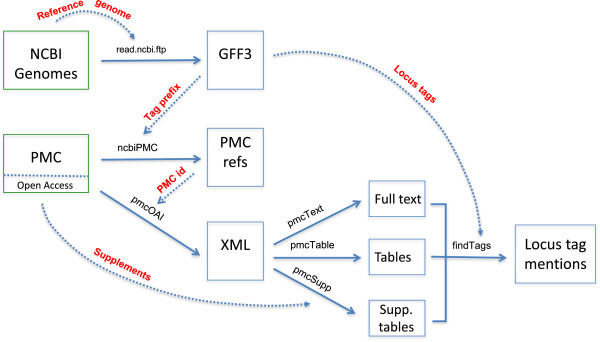
**Flowchart for mining locus tags using the pmcXML package.** R functions are indicated by solid lines, inputs by dash lines, and NCBI databases and R objects by boxes. For each species, NCBI Genomes is used to find the reference strain and download the GFF3 file. The locus tag prefixes in the GFF3 files are used to format a search query in PubMed Central and find matching references. For each reference, the PMC id is used to download the XML document which is then parsed into full text and tables. The XML file includes links to supplements that are downloaded separately, but typically require additional code to reformat (therefore, only locus tags within supplements from *B. pseudomallei* were extracted). Finally, the locus tags are used to create a pattern string to extract tags and also expand locus tag pairs marking the start and end of a region. The R functions developed specifically for this effort are described in Additional file 2.

Searching for all locus tags in PMC requires other steps that are outlined in the flowchart in Figure 1. We selected ten species (Table 1) and searched Entrez Genome to find the strain listed as the “Reference genome, Community selected”. We downloaded GFF3 files from the NCBI Genomes FTP site to retrieve an ordered list of locus tag identifiers from all features including CDS, pseudogenes and RNAs.

**Table 1:** **Reference genome codes**, **strains and locus tag prefixes used for searching PubMed Central**

**Code**	**Strain**	**Tag prefix**	**RefSeq acc**
BPS	Burkholderia pseudomallei K96243	BPSL, BPSS	NC_006350, NC_006351
Cj	Campylobacter jejuni subsp. jejuni NCTC 11168	Cj	NC_002163
CT	Chlamydia trachomatis D/UW-3/CX	CT	NC_000117
FTT	Francisella tularensis subsp. tularensis SCHU S4	FTT	NC_006570
HP	Helicobacter pylori 26695	HP	NC_000915
lmo	Listeria monocytogenes EGD-e	lmo	NC_003210
Rv	Mycobacterium tuberculosis H37Rv	Rv	NC_000962
PA	Pseudomonas aeruginosa PAO1	PA	NC_002516
VC	Vibrio cholerae O1 biovar El Tor str. N16961	VC, VCA	NC_002505, NC_002506
YPO	Yersinia pestis CO92	YPO	NC_003143

We used the locus tag prefix and first digit from the GFF3 file to build wildcard searches and find PMC articles with a matching locus tag (Additional file 1: Table S1). We also restricted the number of spurious matches by limiting the results to articles with the genus name in the title or abstract. For example, this query was used for *Yersinia pestis* CO92: (YPO0* OR YPO1* OR YPO2* OR YPO3* OR YPO4*) AND (Yersinia [ABSTRACT] OR Yersinia[TITLE]) AND open access[FILTER]. In some cases, the results returned a warning that the wildcard search used only the first 600 variations and therefore we lengthened the root word to include two digits.

For each publication, we passed the PMC id to the Open Archives Initiative service and downloaded the XML version of the full text article. We parsed the XML into text by splitting the document into main sections, and each section was further divided into complete sentences. We parsed XML tables using rowspan and colspan attributes to correctly position and repeat cell values and then joined column names and cell values into a single delimited list to preserve the table structure in a single row. We extracted tags from both main text and tables by matching a prefix followed by four digits (or three digits in *Chlamydia*) and optional suffixes. We then expanded locus tag pairs marking the start and end of a region such as an operon or genomic island using the ordered list of tags in the GFF3 file. We saved the PMC id, locus tag, section title or table caption, full sentence or table row, and a flag indicating if the tag was mentioned indirectly within a range. We manually checked all ranges with ten or more locus tags to ensure valid range expansions. Finally, we searched for additional locus tags in *B. pseudomallei* from supplementary tables and from full text articles outside the OA subset.

A complete description of the R functions listed in Figure 1 is available in the supplementary text in Additional file 2. The R code is also available on GitHub (https://github.com/cstubben/pmcXML) for further community development.

## Results

### Locus tags in reference genomes

**Figure 2:**
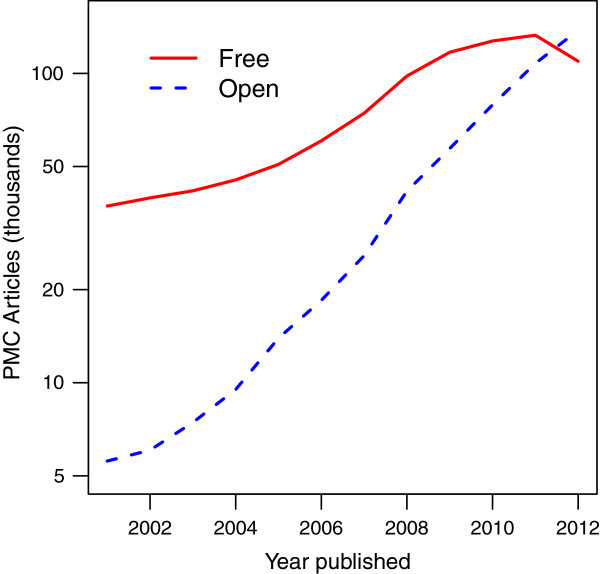
Total number of articles published each year in the rapidly growing Open Access subset that are available for text mining compared to other PMC articles that are only free to read.

There are over 2.88 million articles in PMC and 681,814 articles (23.6%) are included in the Open Access subset (accessed Nov 5, 2013). The number of OA publications is increasing rapidly each year (Figure 2) and 56% of PMC articles published in 2012 are open and available for text mining.

We searched for articles from ten microbial genomes (Table 1) with locus tag mentions by using the locus tag prefix as a wildcard pattern and found 3011 total articles in PubMed and 9282 articles in PMC, ranging from 123 articles in *Burkholderia pseudomallei* to 3569 publications in *Pseudomonas aeruginosa* (Additional file 1: Table S2). In order to find the most relevant articles in PMC, we also matched the genus name in the title or abstract and limited the results to the OA subset, which ranged from 35 articles in *Yersinia pestis* to 693 in *Mycobacterium tuberculosis*.

We downloaded the XML documents from 1835 OA publications and extracted locus tags within the full text and tables (Table 2). The wildcard searches returned 389 papers without a locus tag, simply because the truncated pattern required by PMC returns the papers while the extraction step fails to return a valid locus tag string. Most of these articles matched a strain name sharing the same prefix as a locus tag (e.g., strain PA14); more problematic are strain names like VC3477 and VC4370 in *Vibrio cholerae* that also match a valid tag name and are false positives in the results.

**Table 2:** Total number of open access articles with locus tag mentions by source

			**Direct mentions**		**Within range**	
**Code**	**Articles**	**Mentions**	**Text**	**Tables**	**Text**	**Tables**
BPS	53	3675	832	682	1379	782
Cj	85	4193	2094	1116	654	329
CT	66	3268	1841	950	476	1
FTT	54	1709	603	1027	79	0
HP	136	5035	2701	1954	271	109
lmo	65	4227	2470	1144	517	96
Rv	626	26329	13352	7903	4079	995
PA	225	11454	5372	3754	1855	473
VC	102	2633	1450	609	511	63
YPO	34	1900	176	1350	183	191

We identified 30,891 locus tags in main text and 20,489 tags in tables (Table 2). We expanded locus tag ranges and identified another 13,043 tags mentioned indirectly within a range. The complete list of all 1446 publications and 64,423 tag mentions are available by genome in Additional file 3: Table S3-S13. The majority of tags in *B. pseudomallei* were part of ranges (59%), while the number of tags mentioned indirectly within other genomes included 5% from *Francisella*, 8% from *Helicobacter*, 14% from *Chlamydia* and *Listeria*, and 20-23% from the other five genomes.

We corrected 21 matches to locus tag pairs that were not part of a valid region (Additional file 4: Table S14). Most matches were to interaction pairs such as Rv2158c-Rv0631c from PMC2649132: “Some edges in the SOS response (e.g. Rv2158c-Rv0631c) were common to paths from cell wall proteins and gyrase”. Other matches included network paths, primer names, comparisons and ranges spanning the origin of replication such as Rv3913-Rv0017c. In this case, the parser returned 4082 tags between Rv0017c and Rv3913 instead of the 25 tags between Rv3913 and Rv0017c. We also corrected six large range expansions that were the result of typographical errors in the published articles. We noted a few cases where ranges should be expanded but the current parser did not detect them automatically, for example, some tables list the start and end of a region in different columns in a table.

We matched 13,642 unique locus tags to identifiers in the RefSeq GFF3 files (Table 3), which is 42% of all RefSeq genes in the ten genomes. For comparison, the Entrez Gene database contains only 1609 genes linked to PubMed articles (Additional file 1: Table S2). The locus tag mining provided eight times as many gene-literature links than currently available. We identified 731 putative locus tags that were not found in the RefSeq GFF3 files. Almost all the putative tags were due to the inconsistent use of a “c” suffix on the minus strands in strains of *Campylobacter*, *Francisella* and *Mycobacterium* and could be resolved. In a few cases, new locus tags such as BPSS3220 in *B. pseudomallei* were identified but not found in any gene database. Overall, the total number of locus tags with mentions in the OA subset ranged from 19% in *V. cholerae* to 82% in *M. tuberculosis*.

**Table 3:** **Total number of unique locus tags in RefSeq**, **PMC and in both databases and the source of the unique locus tag**

	**Unique locus tags in**			**Unique tags mentioned in**		
**Code**	**RefSeq**	**Both**	**PMC**	**Text**	**Both**	**Range**
BPS	5935	1575	1588	466	194	928
Cj	1699	863	1009	683	196	130
CT	940	620	626	243	183	200
FTT	1852	687	792	740	49	3
HP	1627	928	977	770	149	58
lmo	2940	1092	1094	845	160	89
Rv	4111	3354	3686	2030	1293	363
PA	5571	2488	2507	1853	470	184
VC	4007	766	803	535	95	173
YPO	4087	1269	1291	994	134	163

### Locus tags in *Burkholderia pseudomallei*

In order to better estimate the fraction of locus tags indexed by OA publications, we also checked supplementary tables and other publications in *B. pseudomallei* (Additional file 5: Table S15-S19). There were 53 Open Access articles in PMC containing 1514 direct mentions and 2161 tags within ranges (3675 total). There were another 53 free articles in PMC with 1514 direct mentions and 1304 tags within ranges (2818 total). There were 16 articles in PMC matching the locus tag and the genus name *Burkholderia* anywhere in the full text. These articles included very few mentions as expected (52 total) and over half the tags were from two tables listing type VI secretion system homologs in *B. pseudomallei*. We also identified seven PMC articles not found in the search results, including five with tags in supplementary tables only. For example, the study by Schell et al. [13] lists 653 virulence genes in a zipped document file in the supplement, so these virulence genes are not even available using web searches.

The 63 supplementary tables contained the majority of all locus tags (83%) and included 40,122 total mentions from 30 publications. The supplements included 21 Word tables, 19 PDF tables, 14 Excel files, four HTML tables, three zipped files and two GenBank files (Additional file 6: Table S20). The pmcSupp function in the R package was used to read all file types directly into data frames in R, except for PDF tables that were loaded as a vector of text and required additional code to reformat the table structure. We included the GenBank files from the genome reannotation [14] since these 6263 locus tags included operon groups, novel proteins and revised start coordinates for 1579 proteins.

Finally, we searched PubMed for any articles not included in PMC. We identified 14 PubMed articles matching the *B. pseudomallei* locus tag prefix in the abstract. Nine of these articles have the full text available from the publisher and we extracted 390 mentions. We found another 25 PubMed articles containing 1382 total mentions from our own reference collection, although there are likely many other publications in this group that have not been identified. Overall, we retrieved 168 total articles and extracted 48,470 total mentions (Additional file 5: Table S15-S19).

There were 22 *B. pseudomallei* locus tags mentioned in 15 or more publications (Table 4). Seven of these proteins are annotated as hypothetical proteins in Entrez Gene and another three are marked as putative proteins in UniProt, so nearly half of the highly cited proteins are hypothetical or putative proteins in one of these two major protein databases. A few of the hypothetical proteins are reviewed below. BPSS1492 is mentioned in 22 different publications and was first designated in 2005 as the *Burkholderia* intracellular motility A protein or BimA, which is required for actin-based motility [15]. BPSL1549 is the *Burkholderia* Lethal Factor 1 (BLF1) and includes a known structure in the Protein Data Bank (PDB) [16]. BPSL1705 and BPSS0796 are *Burkholderia* Oca-like adhesin proteins or BoaB and BoaA [17]. BPSS1434 is named the *Burkholderia pseudomallei* adhesion A protein or BpaA [18]. It was noted by Adler et al. that the new annotation gives BPSS1434 that same gene name as an unrelated Type V two-partner secreted BpaA found in some Australian strains of *B. pseudomallei*[19]. BPSS1385 is a homolog of cycle inhibiting factors (CifBp) and also has a structure in PDB [20]. BPSS1498 is part of the type VI secretion system and included in a large gene cluster BPSS1496-BPSS1511 [21,22] with two other hypothetical proteins in Table 4.

**Table 4:** **Total number of articles citing a *B. pseudomallei *** **locus tag and the number of times each tag was mentioned directly within the text or indirectly within a range**

		**Mentions**		
**Locus tag**	**Articles**	**Direct**	**Range**	**RefSeq definition**
BPSS1492	22	36	1	Hypothetical protein
BPSL1549	21	94	2	Hypothetical protein
BPSL2697	21	41	1	Molecular chaperone GroEL
BPSL1705	19	47	7	Hypothetical protein
BPSS0796	18	42	0	Surface-exposed protein
BPSS1434	18	53	2	Membrane-anchored cell surface protein
BPSS1529	18	19	6	Membrane antigen
BPSS1532	18	23	9	Cell invasion protein
BPSS2288	18	32	0	Heat shock protein 20
BPSS1385	17	17	5	ATP/GTP binding protein
BPSS1545	17	15	7	Type III secretion system protein
BPSL2522	16	26	0	Outer membrane protein a
BPSS0421	16	24	7	Lipopolysaccharide biosynthesis protein
BPSS1498	16	23	13	Hypothetical protein
BPSS1531	16	14	8	Cell invasion protein
BPSS1546	16	15	7	AraC family transcriptional regulator
BPSL3319	15	33	2	Flagellin
BPSS1509	15	29	14	Hypothetical protein
BPSS1511	15	22	5	Hypothetical protein
BPSS1539	15	27	5	Hypothetical protein
BPSS1542	15	12	5	Surface presentation of antigens protein
BPSS1544	15	11	7	Type III secretion system protein

## Discussion

Since the number of OA publications is increasing rapidly (Figure 2), tools that automatically link gene identifiers to recent articles in full text databases could improve microbial gene annotation in many ways. Within the document, the locus tags could be highlighted and linked to protein databases. Within a protein database, the links to publications containing the locus tag and the specific sentence or table row could be provided, along with the context of the mention such as a section title or table caption (see Additional file 3: Table S3-S13 and Additional file 5: Table S15-S19 for all mentions containing locus tags). The mentions could also be viewed as tracks in genome browsers or processed further to summarize structural and functional annotations.

Structural annotations include revised gene starts, novel genes, doubtful coding regions and mobile regions. In *B. pseudomallei*, over half of RefSeq genes have alternate starts in the Genemark, Glimmer or Prodigal predictions available in the same genomes FTP directory at NCBI. Therefore, finding verified start coordinates in the primary literature based on either experimental or computational evidence would be very useful. Prodigal has been recommended for *Burkholderia* genomes due to their high GC content, and Dunbar et al. [23] includes gene start revisions for 994 inconsistent ortholog sets. However, the locus tags and coordinates reported for *B. pseudomallei* are from strain 1710b. Since many of these protein sequences have 100% similarity to the corresponding protein sequences of K96243, locus tags in closely related strains would be another valuable resource to improve annotations. In addition, the *B. pseudomallei* genome reannotation by Nandi et al. [14] included 1579 RefSeq proteins with new start coordinates.

Other useful sources of structural annotations in the primary literature include novel genes and doubtful coding regions. The reannotation by Nandi et al. identified 283 novel genes and 120 doubtful CDSs in the supplementary tables [14]. One of the novel genes included BPSL1057F1 and the protein reportedly increased actin stress fiber formation in transfected cells. Since these novel genes are only found in the literature, they are often missed by tagging systems based solely on dictionary lookups. We extracted locus tags based on pattern searches, which returned 731 additional tags not found in the RefSeq GFF3 files (Table 3).

Functional annotations include gene names, definitions and less often terms from controlled vocabularies describing functions and other characteristics. Protein definitions and gene names are critical for comparative analyses since they are the most commonly used source of information transfer [1]. Clearly, public sequence databases and annotation service providers have failed to keep up with the increasing number of publications, and as illustrated in Table 4, many commonly cited locus tags are still listed as hypothetical proteins. For example, BPSS1492 is mentioned in 22 different publications and was first identified as a *Burkholderia* intracellular motility A protein (BimA) from Stevens et al. in 2005 [15]. There are also 47 papers in PMC matching BimA and *Burkholderia*; however, the gene name *bimA* is not included in any public sequence database for strain K96243 including NCBI, UniProt and Ensembl. This gene name is also missing from annotations provided by IMG [24], RAST [25], and specialized databases, such as PATRIC [26] or Burkholderia.com [27] as well as the reannotation by Nandi et al. [14]. In 2004, the National Institute of Allergy and Infectious Diseases funded eight Bioinformatic Resource Centers to provide access to pathogen genomes [28]. As part of this effort, curated *Burkholderia* annotations were available from the now defunct Pathema database at JCVI [29] and BimA was correctly identified in this database. However, these were not propagated to the other databases.

In this study, we focused only on locus tags. However, there are many other gene identifiers that should be extracted. In fact, many tables and text sources list gene names by default, and only use a locus tag if a gene name was not assigned by RefSeq or other annotation source. For example, this sentence in Bartpho et al. [30] is typical: “Further confirmation of the presence of some selected virulence genes; FliC, bsaQ, rpoS, BPSL2800, BPSS0120, BPSL1705 and BPSS2053 was also performed”. The gene names *fliC*, *bsaQ*, and *rpoS* correspond to BPSL3319, BPSS154, and BPSL1505 respectively in the RefSeq GFF3 file; therefore, extracting gene names from OA publications will definitely improve microbial genome annotations. In an effort to obtain the most accurate annotations for *B. pseudomallei* genomes, we are continuing to develop R scripts to extract these gene names.

There are many challenges in extracting gene identifiers from the literature, and some groups like the UCSC Genocoding project are actively trying to mine articles outside the OA subset to expand access to human gene and sequence mentions [31]. At least for microbial genomes, and *B. pseudomallei* in particular, we believe that freely available supplementary materials and locus tags mentioned indirectly within ranges are important sources for acquiring gene annotations. Other sources including gene names, accession numbers and coordinates should also be collected before proceeding with future efforts to summarize functions, interactions and pathways.

## Conclusions

Only 1514 *B. pseudomallei* locus tags are mentioned directly in the main text and tables of the OA subset and are indexed and available for searching in PMC. This represents 3% of the total number of *B. pseudomallei* locus tags mentioned in the literature, since most locus tags are available in supplementary tables or within ranges. Both of these are valuable annotation sources and we developed queries and tools in the pmcXML package to improve access to these data sources.

Due to the rapid growth of OA submissions, extracting gene and locus tags from the literature would clearly benefit efforts to improve microbial genome annotation. The next challenge will involve developing the data mining algorithms needed to automatically summarize the gene mentions to identify names and functions of experimentally characterized proteins such as virulence factors and antibiotic resistance genes directly from the literature database. If successful, this would help to convert a full text database into a functional gene annotation database first envisioned by Bourne [32], and would provide a valuable reference for most microbial genomes that do not have recent or updated annotations available in public sequence databases.

## Availability and requirements

**Project name:** pmcXML

**Project home page:**https://github.com/cstubben/pmcXML

**Operating system(s):** Platform independent, however loading supplementary tables requires a number of Unix dependencies (unzip, unoconv, pdftotext) to read zip, Word tables and pdf files

**Programming language:** R

**Other requirements:** Package dependencies include stringr and gdata from CRAN, genomes from Bioconductor and genomes2 from GitHub

**License:** GPL

**Any restrictions to use by non-academics:** none

## Competing interests

The authors declare that no competing interests exist.

## Authors’ contributions

CJS wrote the R code to mine locus tags and wrote the manuscript. JFC defined the data mining task applied to *B. pseudomallei* genomes and edited the manuscript. Both authors read and approved the final manuscript.

## Supplementary Material

Additional file 1: Table S1-S2 Summary of NCBI searches in PubMed and PMC using locus tag prefixes and a summary of Gene-PubMed links in the Entrez Gene database. Click here for file

Additional file 2 A description of R functions in the pmcXML package and R code used to parse locus tags. Click here for file

Additional file 3: Table S3-S13 A list of 1446 publications from ten reference genomes and 64,423 locus tag mentions in the PMC Open Access subset. Click here for file

Additional file 4: Table S14 Locus tag pairs detected as ranges that should not be expanded. Click here for file

Additional file 5: Table S15-S19 A list of 168 publications from *Burkholderia pseudomallei* K96243 and 48,470 locus tags mentions in the scientific literature. The publications include counts of locus tags in full text, tables or supplements. Click here for file

Additional file 6: Table S20 A list of 63 supplementary tables from *Burkholderia pseudomallei* with counts of locus tags mentions, file types and formatting notes. Click here for file
