# Supplementary material for: Mining locus tags in PubMed Central to improve microbial gene annotation
Source: BMC Bioinformatics. 2014 Feb 5;15:43. doi: 10.1186/1471-2105-15-43 (PMC3937057; doi:10.1186/1471-2105-15-43)
Supplement: Additional file 2 — A description of R functions in the pmcXML package and R code used to parse locus tags. [file 1471-2105-15-43-S2.doc]

**Supplementary text**

The functions in the pmcXML package are reviewed below and code to download mentions from ten reference genomes is listed in the last section. The pmcXML package is currently available on GitHub for further community development (https://github.com/cstubben/pmcXML).

*Install packages*

To install the package, first install the required dependencies, stringr and gdata from CRAN, genomes from Bioconductor and genomes2 from github.

install.packages("stringr")

install.packages("gdata")

source("http://bioconductor.org/biocLite.R")

biocLite("genomes")

library(devtools)

install_github("cstubben/genomes2")

install_github("cstubben/pmcXML")

*Download Reference Genomes*

There are over 30,000 locus tag prefixes registered at NCBI (http://www.ncbi.nlm.nih.gov/genomes/lltp.cgi) and over 150 microbial species have 20 or more sequenced strains with a different locus tag prefix. In many cases, it is necessary to identify the strain that is most often studied in the literature and in Entrez Genomes these are often listed as the Reference genome or Community selected. For example, the *Burkholderia pseudomallei* (http://www.ncbi.nlm.nih.gov/genome/476) page in Entrez Genomes lists the Reference genome as *B. pseudomallei* strain K96243. This strain may also be identified using the referenceGenome function in the genomes2 package, which searches Entrez Genome using a species name. The next step is to download the annotations from the Genomes FTP site (ftp.ncbi.nlm.nih.gov/genomes/Bacteria). The read.ncbi.ftp function in genomes package requires the name of the FTP organism directory (usually a combination of name and project id) and reads most types of RefSeq files on the site including GFF3 files below. The final command saves the ordered list of locus tags.

referenceGenome("Burkholderia pseudomallei")

[1] "Reference genome, Community selected, UniProt : Burkholderia pseudomallei K96243"

[2] "Project id : 57733"

data(Bacteria) # list of directories in FTP

subset(Bacteria, pid == 57733)

name mode size date pid

386 Burkholderia_pseudomallei_K96243_uid57733 d 4096 2010-12-06 57733

bpgff <- read.ncbi.ftp( "Burkholderia_pseudomallei_K96243_uid57733", "gff")

bpgff

GRanges with 5935 ranges and 4 metadata columns:

seqnames ranges strand | locus feature description gene

<Rle> <IRanges> <Rle> | <character> <character> <character> <character>

[1] NC_006350 [ 1, 1116] - | BPSL0001 CDS carboxylate-amine ligase

[2] NC_006350 [1161, 2375] - | BPSL0002 CDS hypothetical protein

...

table(values(bpgff)$feature)

CDS miscRNA pseudo rRNA tRNA

5728 8 126 12 61

bplocus <- values(bpgff)$locus

The summaryTag function in genomes2 lists the locus tag prefixes, suffixes and tag ranges from coding regions. The prefixes are needed to search PMC, create the string pattern to extract locus tags from XML, and expand tag pairs marking the start and end of a region. Alternately, the locus tags or gene names could be used as a dictionary to find matches within the document, but in many cases there are new locus tags and gene names in the literature that are not found within GFF3 files.

summaryTag(bpgff)

$prefix

BPSL BPSS

3399 2329

$suffix

a A b B c d

36 42 3 6 1 1

$range

[1] 1 3431

$digits

4

5728

*Find publications*

The next step is to find relevant publications containing any *B. pseudomallei* K96243 locus tag. To find all full-text articles with a possible locus tag, we use the tag prefix and first digit from the GFF3 file to build a wildcard search. We restrict the number of spurious matches by limiting the results to articles with the genus name in the abstract or title and articles in the OA subset since these are available for text-mining as XML.

tags <- "(BPSL0* OR BPSL1* OR BPSL2* OR BPSL3* OR BPSS0* OR BPSS1* OR BPSS2*)"

bp <- ncbiPMC(paste(tags, "AND (Burkholderia[TITLE] OR Burkholderia[ABSTRACT]) AND open access[FILTER]"))

bp[1:10,]

pmc authors year title journal volume pages epubdate pmid

1 PMC3623717 Hara Y, Chin CY, Mohamed R, et al 2013 Multiple-antigen ELISA for melioidosis - a novel approach to the improved serodiagnosis of melioidosis BMC Infectious Diseases 13 165 2013/04/04 23556548

2 PMC3607239 Puah SM, Puthucheary S, Chua KH 2013 Potential Immunogenic Polypeptides of Burkholderia pseudomallei Identified by Shotgun Expression Library and Evaluation of Their Efficacy International Journal of Medical Sciences 10 539-547 2013/03/13 23532805

3 PMC3579680 Janse I, Hamidjaja RA, Hendriks AC, et al 2013 Multiplex qPCR for reliable detection and differentiation of Burkholderia mallei and Burkholderia pseudomallei BMC Infectious Diseases 13 86 2013/02/14 23409683

4 PMC3564208 Choh LC, Ong GH, Vellasamy KM, et al 2013 Burkholderia vaccines: are we moving forward? Frontiers in Cellular and Infection Microbiology 3 5 2013/02/05 23386999

5 PMC3540353 Dowling AJ 2013 Novel gain of function approaches for vaccine candidate identification in Burkholderia pseudomallei Frontiers in Cellular and Infection Microbiology 2 139 2013/01/09 23316481

6 PMC3527420 Chen R, Barphagha IK, Karki HS, et al 2012 Dissection of Quorum-Sensing Genes in Burkholderia glumae Reveals Non-Canonical Regulation and the New Regulatory Gene tofM PLoS ONE 7 e52150 2012/12/20 23284909

7 PMC3521395 Khoo JS, Chai SF, Mohamed R, et al 2012 Computational discovery and RT-PCR validation of novel Burkholderia conserved and Burkholderia pseudomallei unique sRNAs BMC Genomics 13 S13 2012/12/07 23282220

8 PMC3443583 Ong HS, Mohamed R, Firdaus-Raih M 2012 Comparative Genome Sequence Analysis Reveals the Extent of Diversity and Conservation for Glycan-Associated Proteins in Burkholderia spp Comparative and Functional Genomics 2012 752867 2012/09/06 22991502

9 PMC3419357 Burtnick MN, Heiss C, Roberts RA, et al 2012 Development of capsular polysaccharide-based glycoconjugates for immunization against melioidosis and glanders Frontiers in Cellular and Infection Microbiology 2 108 2012/08/15 22912938

10 PMC3418162 Chieng S, Carreto L, Nathan S 2012 Burkholderia pseudomallei transcriptional adaptation in macrophages BMC Genomics 13 328 2012/07/23 22823543

*Download PMC XML*

The XML version of Open Access articles are downloaded from the Open Archives Initiative (OAI) service using the pmcOAI function. This function also adds carets (^) within superscript tags and hyperlinked table footnotes for displaying as plain text (since numeric footnotes are often associated with numeric values or character footnotes are added to ends of locus tags, for example, BPSL0075<sup>a</sup> is displayed as BPSL0075^a and not BPSL0075a since both BPSL0075 and BPSL0075a are valid tag names). The function also saves a local copy for future use and will use that copy instead of downloading a second time. Finally, the function uses the xmlParse function from the XML (<http://cran.r-project.org/web/packages/XML/index.html>) package to read the file and generate the XML tree within the R session, so objects are stored as an XMLInternalDocument class and can be queried using XPath expressions. In this example, the last reference in the list above from Chieng et al 2012 (<http://www.ncbi.nlm.nih.gov/pmc/articles/PMC3418162>) is loaded into R using the OAI service.

id <- "PMC3418162"

doc <- pmcOAI(id)

A number of different XPath queries can be used to explore the XML document content and a few are described below including xpathSApply and getNodeSet. This query list all 87 tags and counts the number of occurrences.

table( xpathSApply(doc, "//*", xmlName))

abstract aff article article-categories

1 2 1 1

article-id article-meta article-title back

4 1 62 1

body bold caption col

1 40 13 13

colgroup contrib contrib-group copyright-holder

3 3 1 1

copyright-statement copyright-year date datestamp

1 1 2 1

day email ext-link fig

3 3 7 8

fpage front GetRecord given-names

62 1 1 363

graphic header history hr

8 1 1 220

identifier issn issue italic

1 1 43 188

journal-id journal-meta journal-title journal-title-group

2 1 1 1

kwd kwd-group label license

3 1 13 1

license-p lpage media metadata

1 56 1 1

mixed-citation month name OAI-PMH

61 3 363 1

p permissions pub-date pub-id

57 1 2 107

publisher publisher-name record ref

1 1 1 61

ref-list request responseDate sec

1 1 1 27

self-uri setSpec source sub

1 2 61 7

subject subj-group sup supplementary-material

1 1 14 1

surname table table-wrap table-wrap-foot

363 3 3 1

tbody td th thead

3 232 14 3

title title-group tr underline

28 1 55 4

volume xref year

62 106 65

You can search down the XML tree and find the three main nodes of a PMC XML file. These include the front with abstract, body with main text, and back with references.

xpathSApply(doc, "//article/child::node()", xmlName)

[1] "front" "body" "back"

In some cases, tag names are not specific, so searching up the tree may help find a specific type of tag. For example, article-titles are included within the references or main title and therefore both tags are needed to return the main title.

table( xpathSApply(doc, "//article-title/parent::node()", xmlName) )

mixed-citation title-group

61 1

xpathSApply(doc, "//title-group/article-title", xmlValue)

[1] "Burkholderia pseudomallei transcriptional adaptation in macrophages"

Captions may also be associated with figures, tables and supplements, so listing only table captions requires adding the table-wrap node before the caption.

table( xpathSApply(doc, "//caption/parent::node()", xmlName) )

fig media supplementary-material table-wrap

8 1 1 3

xpathSApply(doc, "//table-wrap/caption", xmlValue)

[1] "Twenty-five common up-regulated genes of B. pseudomallei during intracellular growth in host macrophages relative toin vitro growth"

[2] "Gene function enrichment analysis of B. pseudomallei common up-regulated and down-regulated genes throughout growth within host macrophages"

[3] "List of oligonucleotides used in real-time qPCR experiments"

The first function below will list all 27 section titles and the second functions lists only the 8 main sections in the document (not subsections). The pmcXML package uses this XPath query to split the main document into sections using getNodeSet and then loops through each section to split the full text into complete sentences.

xpathSApply(doc, "//sec/title", xmlValue)

xpathSApply(doc, "//body/sec/title", xmlValue)

[1] "Background" "Results" "Discussion" "Conclusions" "Methods" "Competing interests"

[7] "Authors’ contributions" "Supplementary Material"

x <-getNodeSet(doc, "//body/sec")

x[[1]]

*Parse XML*

The pmcXML package includes three functions to parse full-text, tables and supplements from the XML document (pmcText, pmcTable, pmcSupp). The pmcText function splits the XML document into main sections and also includes title, abstract, section titles, and captions from figure, table and supplements (references are optional). In addition, the text within paragraph tags in each section is split into complete sentences by taking care to avoid splitting after genus abbreviations like *E. coli* or other common abbreviations such as Fig., et al., e.g., i.e., sp., ca., vs., and many others (an option is included to split sentences using sentDetect in the openNLP package if installed). In this example, the sapply function is used to count the number of sentences in each section.

unlist(xpathSApply(doc, "//article", xmlValue))

x1 <- pmcText(doc)

sapply(x1, length)

Main title Abstract Background Results

1 8 21 77

Discussion Conclusions Methods Competing interests

52 3 68 1

Authors’ contributions Section title Figure text Table caption

4 27 37 5

Supplement caption

1

x1[1:2]

Main title

[1] "Burkholderia pseudomallei transcriptional adaptation in macrophages."

$Abstract

[1] "Burkholderia pseudomallei is a facultative intracellular pathogen of phagocytic and non-phagocytic cells."

[2] "How the bacterium interacts with host macrophage cells is still not well understood and is critical to appreciate the strategies used by this bacterium to survive and how intracellular survival leads to disease manifestation."

[3] "Here we report the expression profile of intracellular B. pseudomallei following infection of human macrophage-like U937 cells."

[4] "During intracellular growth over the 6 h infection period, approximately 22 % of the B. pseudomallei genome showed significant transcriptional adaptation."

[5] "B. pseudomallei adapted rapidly to the intracellular environment by down-regulating numerous genes involved in metabolism, cell envelope, motility, replication, amino acid and ion transport system and regulatory function pathways."

[6] "Reduced expression in catabolic and housekeeping genes suggested lower energy requirement and growth arrest during macrophage infection, while expression of genes encoding anaerobic metabolism functions were up regulated."

[7] "However, whilst the type VI secretion system was up regulated, expression of many known virulence factors was not significantly modulated over the 6hours of infection."

[8] "The transcriptome profile described here provides the first comprehensive view of how B. pseudomallei survives within host cells and will help identify potential virulence factors that are important for the survival and growth of B. pseudomallei within human cells."

The list can also be searched directly using the grep function and since these types of searches are common, we created a wrapper called searchP that returns the results as a single table. The findTags, findGenes and other functions described in the next section also use searchP to find matches.

lapply(x1, function(y) grep( "BPS[SL]", y, value=TRUE) )

searchP(x1, "BPS[SL]")

section mention

1 Results Anaerobic metabolism pathway genes such as BPSS1279 (threonine dehydratase), BPSL1771 (cobalamin biosynthesis protein CbiG) and BPSS0842 (benzoylformate decarboxylase) were up-regulated throughout the infection period.

2 Results Nevertheless, none of the components of the anaerobic respiratory chain showed significant changes in expression except for BPSL2311 (putative respiratory nitrate reductase delta chain) and BPSL2312 (putative respiratory nitrate reductase gamma chain) that were induced at the early stage of infection.

3 Results The major nitrogen source in the intracellular compartment is most likely methylamine and purine as suggested by the increased expression of methylamine utilization protein (BPSS0404) and allantoicase (BPSL2945).

4 Results These include the main capsular polysaccharide biosynthesis (BPSL2787-BPSL2810) genes, two potential surface polysaccharide biosynthesis gene clusters (BPSS0417-BPSS0429 and BPSS1825-BPSS1834), majority of genes in the lipopolysaccharide (LPS) biosynthesis cluster and genes encoding for flagella assembly and chemotaxis.

5 Results One of the six clusters of the type VI secretion system, the tss-5 cluster (BPSS1493-BPSS1511), was up-regulated up to 182-fold during intracellular infection (Figure 8).

6 Results We also observed the induction of genes flanking the tss-5 cluster, bimA (Burkholderiaintracellular motility A)(BPSS1492) and BPSS1512 at 2 to 6 h post-infection.

7 Results Moreover, the hemolysin activator-like protein precursor, fhaC (BPSS1728) gene was significantly up-regulated during intracellular infection.

8 Results Consistently, the large filamentous hemagglutinin precursor, fhaB (BPSS1727) gene, a potential virulence factor of B. pseudomallei[20], was induced between 2 to 6 h post-infection.

9 Discussion In this study, high induction of tssD-5 (BPSS1498), an effector Hcp1 protein of T6SS was observed throughout the infection period.

The pmcTable function parses the XML tables into a list of data.frames. This functions uses rowspan and colspan attributes to correctly format and repeat cell values as needed. For example, Table 1 (http://www.ncbi.nlm.nih.gov/pmc/articles/PMC3418162/table/T1) includes a multi-line header spanning four columns that is repeated across each cell and the two rows are combined into a single header row for display. The caption and footnotes for each table are also saved as attributes.

x2 <- pmcTable(doc)

[1] "Parsing Table 1 Twenty-five common up-regulated genes of B. pseudomallei during intracellular growth in host macrophages relative to in vitro growth"

[1] "Parsing Table 2 Gene function enrichment analysis of B. pseudomallei common up-regulated and down-regulated genes throughout growth within host macrophages"

[1] "Parsing Table 3 List of oligonucleotides used in real-time qPCR experiments"

x2[[1]][1:4, 1:4]

Gene Description Fold Change (in vivo/in vitro) at the indicated time (h): 1 Fold Change (in vivo/in vitro) at the indicated time (h): 2

1 BPSL0184 Putative rod shape-determining protein 23.83 15.31

2 BPSL0842 Benzoylformate decarboxylase 70.27 31.78

3 BPSL0886 Hypothetical protein 12.29 8.36

4 BPSL1067 Hypothetical protein 8.39 5.15

attributes(x2[[1]])

$id

[1] "PMC3418162"

$file

[1] "http://www.ncbi.nlm.nih.gov/pmc/articles/PMC3418162/table/T1"

$label

[1] "Table 1"

$caption

[1] "Twenty-five common up-regulated genes of B. pseudomallei during intracellular growth in host macrophages relative to in vitro growth"

$footnotes

[1] "Note: * Genes selected for real-time qPCR analysis."

Subheadings are common in many tables like Table 2 above (http://www.ncbi.nlm.nih.gov/pmc/articles/PMC3418162/table/T2) and since we often need to display a single row only, these subheadings are repeated down the rows using repeatSub (and will be included as part of pmcTable in future version to automatically repeat subheadings). In addition, we collapse the row into a single delimited string containing column names and row values using collapse2. The searchP function may also be used to search the tables and returns the table name and matching rows in collapsed format.

t2 <- repeatSub(x2[[2]])

t2

subheading Functional class or pathway No. of genes regulated No. of genes in genome Significance (p-value)

1 Up-regulated genes Benzoate degradation via hydroxylation 3 29 3.33 × 10^-2

2 Down-regulated genes Amino sugar and nucleotide sugar metabolism 22 39 7.98 × 10^-10

3 Down-regulated genes Bacterial chemotaxis 23 46 2.65 × 10^-9

collapse2(t2)[1:3]

[1] "subheading=Up-regulated genes;Functional class or pathway=Benzoate degradation via hydroxylation;No. of genes regulated=3;No. of genes in genome=29;Significance (p-value)=3.33 × 10^-2"

[2] "subheading=Down-regulated genes;Functional class or pathway=Amino sugar and nucleotide sugar metabolism;No. of genes regulated=22;No. of genes in genome=39;Significance (p-value)=7.98 × 10^-10"

[3] "subheading=Down-regulated genes;Functional class or pathway=Bacterial chemotaxis;No. of genes regulated=23;No. of genes in genome=46;Significance (p-value)=2.65 × 10^-9"

searchP(x2, "BPS[SL]") # 32 rows

The pmcSupp function parses the list of supplementary files into a data.frame. Since the XML file only includes links to supplements, a file name or row number from the returned list is needed to load the file into R.

pmcSupp(doc)

label caption file type

1 Additional file 1 List of 1259 common down-regulated genes of B. pseudomallei during intracellular growth in host macrophages relative to in vitro growth. 1471-2164-13-328-S1.xls excel

# s1 <- pmcSupp(doc, "1471-2164-13-328-S1.xls")

s1 <- pmcSupp(doc, 1)

nrow(s1)

[1] 1260

head(s1)

Gene Description Fold change (in vivo/in vitro) at the indicated time (h)

1 1.00 2.00 4.00 6.00

2 BPSL0001 Hypothetical protein -4.68 -2.81 -5.56 -3.47

3 BPSL0004 hupA, DNA-binding protein HU-alpha -10.39 -7.77 -9.82 -3.07

4 BPSL0005 Putative cobalamin synthesis protein/P47K -5.69 -3.97 -5.77 -2.98

5 BPSL0006 Hypothetical protein -6.42 -3.98 -7.50 -6.94

6 BPSL0008 gspE, general secretory pathway protein E -6.42 -3.00 -4.74 -4.79

This pmcSupp function reads files in a variety of formats including Excel, Word, HTML, PDF, and zipped files using a variety of Unix tools and other packages (and therefore running on Unix platforms is recommended). Compressed files are automatically unzipped using the Unix unzip command. Excel files are read using the read.xls function in the gdata package. We added some extra code to the Perl function xls2csv.pl within gdata to add carets before superscripts (again, in many cases numeric footnotes are associated with numeric values or character footnotes are added to ends of locus tags). Microsoft Word documents are converted to HTML files using the Universal Office Converter unoconv and then tables within the html files are read using readHTMLtable in the XML package. The tables within HTML files are also loaded using readHTMLtable. PDF files are converted to text using the Unix pdftotext command and the resulting file is read into R as text using readLines. Most supplementary files require some post-processing, for example, fixing the multi-line header missed by read.xls below.

*Find features*

In order to extract locus tags from the searchP results, we use str_extract_all in the stringr package to extract locus tags using the prefix, number of digits and optional suffixes (returned by summaryTags above) as the pattern string.

y <- searchP(x1, "BPS[SL][0-9]{4}")

str_extract_all(y$mention, "BPS[SL][0-9]{4}[abc]?")

[[1]]

[1] "BPSS1279" "BPSL1771" "BPSS0842"

[[2]]

[1] "BPSL2311" "BPSL2312"

[[3]]

[1] "BPSS0404" "BPSL2945"

[[4]]

[1] "BPSL2787" "BPSL2810" "BPSS0417" "BPSS0429" "BPSS1825" "BPSS1834"

In addition, many locus tags are arranged as pairs marking the start and end of a region such as a genomic island or operon. We also extract these pairs and expand the range using seqIds and the ordered list of locus tags from the GFF3 file.

unlist( str_extract_all(y$mention, "BPS[SL][0-9]{4}-BPS[SL][0-9]{4}") )

[1] "BPSL2787-BPSL2810" "BPSS0417-BPSS0429" "BPSS1825-BPSS1834" "BPSS1493-BPSS1511"

seqIds("BPSS0417-BPSS0429", tags=bplocus)

[1] "BPSS0417" "BPSS0418" "BPSS0419" "BPSS0420" "BPSS0421" "BPSS0422" "BPSS0423" "BPSS0424" "BPSS0425" "BPSS0426" "BPSS0427" "BPSS0428" "BPSS0429"

The findTags function extracts tags and expands ranges using the pmcText or pmcTable output. The resulting data.frame includes the PMC id, section, locus tag, flag indicating if tags were indirectly cited within a range, and the mention (sentence or collapsed row).

x <- findTags(x1, bplocus, prefix = "BPS[SL]" , suffix= "[abc]")

[1] "9 matches"

[1] "Expanded 2 matches to 48, 19 tags"

[1] "79 locus tags cited (78 unique)"

x[1:10,]

id source locus range mention

1 PMC3418162 Results BPSS1279 FALSE Anaerobic metabolism pathway genes such as BPSS1279 (threonine dehydratase), BPSL1771 (cobalamin biosynthesis protein CbiG) and BPSS0842 (benzoylformate decarboxylase) were up-regulated throughout the infection period.

2 PMC3418162 Results BPSL1771 FALSE Anaerobic metabolism pathway genes such as BPSS1279 (threonine dehydratase), BPSL1771 (cobalamin biosynthesis protein CbiG) and BPSS0842 (benzoylformate decarboxylase) were up-regulated throughout the infection period.

3 PMC3418162 Results BPSS0842 FALSE Anaerobic metabolism pathway genes such as BPSS1279 (threonine dehydratase), BPSL1771 (cobalamin biosynthesis protein CbiG) and BPSS0842 (benzoylformate decarboxylase) were up-regulated throughout the infection period.

4 PMC3418162 Results BPSL2311 FALSE Nevertheless, none of the components of the anaerobic respiratory chain showed significant changes in expression except for BPSL2311 (putative respiratory nitrate reductase delta chain) and BPSL2312 (putative respiratory nitrate reductase gamma chain) that were induced at the early stage of infection.

5 PMC3418162 Results BPSL2312 FALSE Nevertheless, none of the components of the anaerobic respiratory chain showed significant changes in expression except for BPSL2311 (putative respiratory nitrate reductase delta chain) and BPSL2312 (putative respiratory nitrate reductase gamma chain) that were induced at the early stage of infection.

6 PMC3418162 Results BPSS0404 FALSE The major nitrogen source in the intracellular compartment is most likely methylamine and purine as suggested by the increased expression of methylamine utilization protein (BPSS0404) and allantoicase (BPSL2945).

7 PMC3418162 Results BPSL2945 FALSE The major nitrogen source in the intracellular compartment is most likely methylamine and purine as suggested by the increased expression of methylamine utilization protein (BPSS0404) and allantoicase (BPSL2945).

8 PMC3418162 Results BPSL2787 FALSE These include the main capsular polysaccharide biosynthesis (BPSL2787-BPSL2810) genes, two potential surface polysaccharide biosynthesis gene clusters (BPSS0417-BPSS0429 and BPSS1825-BPSS1834), majority of genes in the lipopolysaccharide (LPS) biosynthesis cluster and genes encoding for flagella assembly and chemotaxis.

9 PMC3418162 Results BPSL2788 TRUE These include the main capsular polysaccharide biosynthesis (BPSL2787-BPSL2810) genes, two potential surface polysaccharide biosynthesis gene clusters (BPSS0417-BPSS0429 and BPSS1825-BPSS1834), majority of genes in the lipopolysaccharide (LPS) biosynthesis cluster and genes encoding for flagella assembly and chemotaxis.

10 PMC3418162 Results BPSL2789 TRUE These include the main capsular polysaccharide biosynthesis (BPSL2787-BPSL2810) genes, two potential surface polysaccharide biosynthesis gene clusters (BPSS0417-BPSS0429 and BPSS1825-BPSS1834), majority of genes in the lipopolysaccharide (LPS) biosynthesis cluster and genes encoding for flagella assembly and chemotaxis.

The pmcXML package includes a few other functions to find species and genes (using italic tags) and we are working on functions to find accessions, sequences and coordinates within the full-text, tables and supplements. In most articles, there are many gene names that are not included in the RefSeq GFF3 file and more work is needed to track down the source of these genes (most are from *B. pseduomallei*, but many gene names cited in the methods may be from other species).

table2(findSpecies(doc))

[1] "Found 96 species mentions"

Total

Burkholderia pseudomallei 91

Burkholderia cenocepacia 2

Bordetella pertussis 1

Burkholderia mallei 1

Caenorhabditis elegans 1

x<- findGenes(doc)

[1] "Found 30 genes"

table(x$gene)

bimA bspR cydB dnaB dnaE dpsA dspA fhaB fhaC graY hrcA katG minD minE oxyR parA parB parC rpoS

2 1 4 1 1 2 1 3 4 1 1 1 1 4 2 1 2 1 3

bpgenes <- sort(unique(unlist( strsplit(values(bpgff)$gene, ",") )))

unique(x$gene[!x$gene %in% bpgenes])

[1] "bspR" "bimA" "fhaC" "fhaB" "dpsA" "graY" "parA" "dspA"

*Finding all tags*

Finally, we created a loop that uses the list of references from ncbiPMC and downloads each XML file and parses the full-text and tables and extracts all matching locus tags. In this case, the locus tag mentions are saved to a file called bp.tab. Currently, the supplements are not included in the loop and these are downloaded separately since some additional code is

needed to reformat tables before extracting tags.

pmcLoop(bp, tags= bpgff, prefix = "BPS[SL]" , suffix= "[abc]", file="BPS.tab")

**R code to reproduce results**

org <-"Burkholderia_pseudomallei_K96243_uid57733"

bpgff <- read.ncbi.ftp( org, "gff")

bp <- ncbiPMC("(BPSL0* OR BPSL1* OR BPSL2* OR BPSL3* OR BPSS0* OR BPSS1* OR BPSS2*) AND (Burkholderia[TITLE] OR Burkholderia[ABSTRACT]) AND open access[FILTER]"))

pmcLoop(bp, bpgff, "BPS[SL]" , "[abc]", file="BPS.tab")

org<- "Campylobacter_jejuni_NCTC_11168___ATCC_700819_uid57587"

cjgff <- read.ncbi.ftp( org, "gff")

cj <- ncbiPMC("(Cj00* OR Cj01* OR Cj02* OR Cj03* OR Cj04* OR Cj05* OR Cj06* OR Cj07* OR Cj08* OR Cj09* OR Cj10* OR Cj11* OR Cj12* OR Cj13* OR Cj14* OR Cj15* OR Cj16* OR Cj17*) AND (Campylobacter[TITLE] OR Campylobacter[ABSTRACT]) AND open access[FILTER]" )

pmcLoop(cj, cjgff, "Cj", "c", "Cj.tab")

org<-"Chlamydia_trachomatis_D_UW_3_CX_uid57637"

ctgff <- read.ncbi.ftp( org, "gff")

ct<-ncbiPMC("(CT00* OR CT01* OR CT02* OR CT03* OR CT04* OR CT05* OR CT06* OR CT07* OR CT08* OR CT09 OR CT1* OR CT2* OR CT3* OR CT4* OR CT5* OR CT6* OR CT7* OR CT8*) AND (Chlamydia[TITLE] OR Chlamydia[ABSTRACT]) AND open access[FILTER]")

pmcLoop(ct, ctgff, "CT", "(a|m|\\.1|\\.2)", "CT.tab", digits=3 )

org<- "Francisella_tularensis_SCHU_S4_uid57589"

ftgff <- read.ncbi.ftp( org, "gff")

ft <- ncbiPMC("(FTT0* OR FTT1* OR FTT_0* OR FTT_1*) AND (Francisella[ABSTRACT] OR Francisella[TITLE]) AND open access[FILTER]")

## some prefixes with underscore

pmcLoop(ft, ftgff, "FTT_?", "c", "FTT.tab")

org<-"Helicobacter_pylori_26695_uid57787"

hpgff <- read.ncbi.ftp( org, "NC_000915.gff")

hp <- ncbiPMC("(HP00* OR HP01* OR HP02* OR HP03* OR HP04* OR HP05* OR HP06* OR HP07* OR HP08* OR HP09* OR HP10* OR HP11* OR HP12* OR HP13* OR HP14* OR HP15* ) AND (Helicobacter[TITLE] OR Helicobacter[ABSTRACT]) AND open access[FILTER]")

# strain J99 uses JHP prefix -this last option skip tags starting with JHP

pmcLoop(hp, hpgff, "HP_?", "[am]", "HP.tab", "J")

org<-"Listeria_monocytogenes_EGD_e_uid61583"

lmgff <- read.ncbi.ftp( org, "gff")

lm<-ncbiPMC("(lmo00* OR lmo01* OR lmo02* OR lmo03* OR lmo04* OR lmo05* OR lmo06* OR lmo07* OR lmo08* OR lmo09* OR lmo10* OR lmo11* OR lmo12* OR lmo13* OR lmo14* OR lmo15* OR lmo16* OR lmo17* OR lmo18* OR lmo19* OR lmo20* OR lmo21* OR lmo22* OR lmo23* OR lmo24* OR lmo25* OR lmo26* OR lmo27* OR lmo28*) AND (Listeria[TITLE] OR Listeria[ABSTRACT]) AND open access[FILTER]")

pmcLoop(lm, lmgff, "lmo", file="lmo.tab")

org<- "Mycobacterium_tuberculosis_H37Rv_uid57777"

mtgff <- read.ncbi.ftp( org, "gff")

mt <- ncbiPMC( "(Rv00* OR Rv01* OR Rv02* OR Rv03* OR Rv04* OR Rv05* OR Rv06* OR Rv07* OR Rv08* OR Rv09* OR Rv10* OR Rv11* OR Rv12* OR Rv13* OR Rv14* OR Rv15* OR Rv16* OR Rv17* OR Rv18* OR Rv19* OR Rv20* OR Rv21* OR Rv22* OR Rv23* OR Rv24* OR Rv25* OR Rv26* OR Rv27* OR Rv28* OR Rv29* OR Rv30* OR Rv31* OR Rv32* OR Rv33* OR Rv34* OR Rv35* OR Rv36* OR Rv37* OR Rv38* OR Rv39*) AND (Mycobacterium[ABSTRACT] OR Mycobacterium[TITLE]) AND open access[FILTER]" )

pmcLoop(mt, mtgff, "Rv", "[abc]", "Rv.tab")

org<- "Pseudomonas_aeruginosa_PAO1_uid57945"

pagff <- read.ncbi.ftp( org, "gff")

pa <- ncbiPMC( "(PA00* OR PA01* OR PA02* OR PA03* OR PA04* OR PA05* OR PA06* OR PA07* OR PA08* OR PA09* OR PA10* OR PA11* OR PA12* OR PA13* OR PA14* OR PA15* OR PA16* OR PA17* OR PA18* OR PA19* OR PA20* OR PA21* OR PA22* OR PA23* OR PA24* OR PA25* OR PA26* OR PA27* OR PA28* OR PA29* OR PA30* OR PA31* OR PA32* OR PA33* OR PA34* OR PA35* OR PA36* OR PA37* OR PA38* OR PA39* OR PA40* OR PA41* OR PA42* OR PA43* OR PA44* OR PA45* OR PA46* OR PA47* OR PA48* OR PA49* OR PA50* OR PA51* OR PA52* OR PA53* OR PA54* OR PA55*) AND (Pseudomonas[TITLE] OR Pseudomonas[ABSTRACT]) AND open access[FILTER]" )

pmcLoop(pa, pagff, "PA", "(\\.1)", file="PA.tab")

org<- "Vibrio_cholerae_O1_biovar_El_Tor_N16961_uid57623"

vcgff <- read.ncbi.ftp( org, "gff")

vc <- ncbiPMC( "(VC00* OR VC01* OR VC02* OR VC03* OR VC04* OR VC05* OR VC06* OR VC07* OR VC08* OR VC09* OR VC10* OR VC11* OR VC12* OR VC13* OR VC14* OR VC15* OR VC16* OR VC17* OR VC18* OR VC19* OR VC2* OR VCA0* OR VCA1*) AND ( Vibrio[ABSTRACT] OR Vibrio[TITLE]) AND open access[FILTER]")

pmcLoop(vc, vcgff, "VCA?", "a", file="VC.tab")

org<- "Yersinia_pestis_CO92_uid57621"

ypgff <- read.ncbi.ftp( org, "NC_003143.gff")

yp <- ncbiPMC( "(YPO0* OR YPO1* OR YPO2* OR YPO3* OR YPO4*) AND (Yersinia[ABSTRACT] OR Yersinia[TITLE]) AND open access[FILTER]" )

pmcLoop(yp, ypgff, "YPO", "a", "YPO.tab" )
